# A Review on the Evolving Role of Radiation Therapy in the Treatment of Locally Advanced Rectal Cancer

**DOI:** 10.3390/curroncol32080443

**Published:** 2025-08-07

**Authors:** Zeinab Dandash, Tala Mobayed, Sally Temraz, Ali Shamseddine, Samer Doughan, Samer Deeba, Zeina Ayoub, Toufic Eid, Bassem Youssef, Lara Hilal

**Affiliations:** 1 Department of Radiation Oncology, American University of Beirut Medical Center, Beirut 1107 2020, Lebanon; zd27@aub.edu.lb (Z.D.); tm47@aub.edu.lb (T.M.); za47@aub.edu.lb (Z.A.); te04@aub.edu.lb (T.E.); 2 Department of Hematology-Oncology, American University of Beirut Medical Center, Beirut 1107 2020, Lebanon; st29@aub.edu.lb (S.T.); as04@aub.edu.lb (A.S.); 3 Department of Surgery, American University of Beirut Medical Center, Beirut 1107 2020, Lebanon; sd65@aub.edu.lb (S.D.); sd08@aub.edu.lb (S.D.)

**Keywords:** locally advanced rectal cancer, total neoadjuvant therapy, non-operative management, omission of RT, short- versus long-course chemoradiation, personalized treatment

## Abstract

Treatment of locally advanced rectal cancer (LARC), clinical stages II–III, typically involves multimodal treatment options. Over the past decade, the role of radiation therapy as a neoadjuvant treatment for LARC has evolved and is currently a part of total neoadjuvant therapy (TNT). Some recently published studies advocate for the omission of radiation therapy entirely, while others report on a non-operative approach that emphasizes the use of higher radiation therapy doses. This review aims to evaluate the latest literature on the current role of radiation therapy in the management of LARC, with a discussion of how to best select the most appropriate treatment protocol based on individual patient and tumor characteristics, comorbidities, and personal needs and preferences.

## Simple Summary

This review discusses the recent developments in the treatment of microsatellite-stable locally advanced rectal cancer (LARC), focusing on the evolving role of radiation therapy as part of total neoadjuvant therapy (TNT). It highlights the importance of incorporating both tumor- and patient-specific factors, along with a thorough discussion regarding individual patient preferences, in determining the best treatment approach. This may range from dose-escalated non-operative radiation therapy to the omission of radiation altogether in select patients.

## 1. Introduction

Colorectal cancer is currently the third most common cancer and the second leading cause of cancer-related deaths worldwide [1]. Rectal cancer has been increasingly recognized as a distinct clinical entity from other colorectal malignancies, both in terms of its diagnosis and the therapeutic approach [2]. The majority of rectal cancers are adenocarcinomas [3]. It is estimated that around 46,950 new cases of rectal cancer will be diagnosed in the USA in 2025 [4], as the rate of rectal cancer seems to be increasing annually in adults younger than 50 years [2].

Rectal cancer is grouped into early-stage (cT1–T2 N0), locally advanced (T3–T4 and/or node-positive), and metastatic (any T any N M1) disease. It is also segregated based on a gene mutation affecting the mismatch repair (MMR) pathway, where patients with deficient MMR (dMMR) who have microsatellite instability (MSI) are known to have an excellent response to immunotherapy, while those with proficient MMR (pMMR), also known as microsatellite-stable (MSS) patients, require a more complicated array of treatments [5,6].

The optimal treatment strategy for locally advanced rectal cancer (LARC) mostly depends on MRI findings, where several factors are used to classify patients into a low or high risk of local recurrence and distant metastasis. High-risk LARC usually involves an MRI-predicted circumferential resection margin (CRM) ≤ 1 mm, advanced T3 substages (T3c/T3d), cT4, extramural vascular invasion (EMVI), cN2 disease, enlarged lateral pelvic lymph nodes, or involved mesorectal fascia (MRF) [7], making it more susceptible to treatment failure or recurrence, which necessitates a more aggressive combination of treatments.

The treatment also depends on the location of the tumor in the rectum, whether it is in the lower (up to 5 cm from the anal verge), middle (from >5 to 10 cm), or upper (from >10 up to 15 cm) third of the rectum [7].

Radiotherapy (RT) has historically played an essential role in LARC since 1990, and despite the many alterations witnessed since, it remains part of today’s gold standard for most patients, especially those with low–mid rectal tumors, whether using a short course of RT, typically delivered as 25 Gy in 5 fractions, or long-course RT, delivered as 45–50.4 Gy in 25–28 fractions.

The current standard of care according to the most recent NCCN guidelines [8] depends on total neoadjuvant therapy (TNT), which consists of a neoadjuvant long course of chemoradiotherapy (CRT) or short-course RT followed or preceded by neoadjuvant chemotherapy. TNT is usually followed either by total mesorectal excision (TME) or surveillance depending on the patient’s clinical response. In select patients with low-risk features, neoadjuvant chemotherapy alone resulting in greater than 20% tumor regression may allow for the omission of radiotherapy, enabling them to proceed directly to surgical resection.

This review focuses on recent updates in the treatment options offered to MSS locally advanced rectal cancer patients, highlighting the role of radiotherapy in each approach.

## 2. Materials and Methods

A comprehensive literature search was conducted using the PubMed, Google Scholar, EMBASE, Cochrane Library, and ClinicalTrials.gov databases. Articles published in English between 28 June 1986 and 3 July 2025 were included. The search strategy included mainly the following keywords: “rectal cancer”, “rectal carcinoma”, “rectal neoplasm”, “colorectal cancer”, “colorectal neoplasm”, “rectal adenocarcinoma”, “locally advanced”, “stage II”, “stage III”, “neoadjuvant therapy”, “preoperative chemoradiotherapy”, “preoperative radiotherapy”, “chemoradiation”, “chemoradiotherapy”, “organ preservation”, “non-operative management”, “watch and wait”, “total mesorectal excision”, and “TME”.

Eligible studies included phase II/III clinical trials, meta-analyses, large cohort studies, and professional guidelines. The evidence was weighted based on the quality of the study design, sample size, and relevance to the review topic. Priority was given to higher levels of evidence such as randomized clinical trials (RCTs), meta-analyses, and systematic reviews. Observational studies were included when RCT data were limited or to provide additional context.

## 3. Radiation Treatment for LARC: From the Past to the Present

The treatment paradigm for LARC began to evolve when several clinical trials conducted in the 1980s and 1990s demonstrated that surgery alone, particularly in advanced cases and in the absence of TME, was associated with significantly high rates of local recurrence [9,10,11]. This is when the superiority of TME, defined as the complete resection of the tumor and the mesorectum with all associated lymph nodes along the avascular embryologic plane, over the conventional surgical techniques was proven in terms of its ability to significantly lower local recurrence while enhancing survival rates, providing an optimal method for rectal cancer resection [12].

A multimodal treatment approach was then established by the NIH Consensus Conference held in 1990, which decided on surgery followed by CRT as the standard of care for LARC, based on robust evidence from key clinical trials, including GITSG 7175, NSABP R-01, and Mayo/NCCTG [9,10,13].

A significant shift in that protocol occurred with the introduction of preoperative short-course radiation therapy, given as 25 Gy in five fractions over one week, which was first demonstrated by the Swedish Rectal Cancer Trial in 1997 [14] that showed better survival rates and locoregional control when compared to those under surgery alone. These outcomes were reinforced by the Dutch Rectal Cancer Trial [15] which also compared preoperative short-course radiotherapy to surgery alone but incorporated mandatory TME as the surgical technique, showing a significant reduction in local recurrence, in addition to a survival benefit in specific subgroups. Short-course RT was added to TME as the new standard of care shortly after.

The next major development was the integration of chemotherapy with radiotherapy, when the EORTC 22921 trial demonstrated the significant benefits of preoperative CRT, given as 45 Gy in 25 fractions with two courses of fluorouracil and leucovorin, in reducing local recurrence rates in LARC after 10 years of follow-up [16].

This CRT regimen offered better results when performed preoperatively, as proven by the German Rectal Cancer Trial [17,18], showing significantly improved locoregional control and toxicity rates when compared to those with postoperative CRT.

Preoperative CRT proved to be comparable to preoperative short-course RT (SCRT), as shown in the Polish II trial [19], where the short-course radiotherapy arm and the CRT arm delivered as 50·4 Gy in 28 fractions of 1·8 Gy with bolus 5-fluorouracil and leucovorin were similar in terms of overall survival (OS), disease-free survival (DFS), local and distant failures, and late complication rates after 8 years of follow-up [20]. This outcome proved that both SCRT followed by immediate surgery and long-course CRT can be part of the standard treatment for LARC, with no superiority of one over the other.

Adjuvant chemotherapy use in LARC was and remains controversial due to potential additive toxicity effect when preceded by radiotherapy, in addition to substantial variation in its effectiveness in clinical practice [21]. Many trials have made it clear that no strong recommendation can be made for its regular use [22,23,24]; instead, it is offered in very specific cases, such as in patients who have undergone partial mesorectal excision (PME) or TME alone or patients who have high-risk features post-surgery [7]. As for the current NCCN guidelines, they recommend adjuvant chemotherapy where resection is contraindicated after TNT [8]. Accordingly, it is best to consider adjuvant chemotherapy for LARC on a case-by-case basis.

In 2018, a retrospective study by Van der Valk et al. [22] drew significant attention to an alternative treatment strategy for rectal cancer—the watch-and-wait (WW) approach—which involves active surveillance instead of TME. This approach was only considered for patients who had achieved a complete clinical response (cCR) following CRT, with the goal of preserving the rectum and minimizing the risks associated with surgical intervention [25]. This retrospective study used an international registry which collected data on more than 1000 patients adopting the WW strategy, yielding impressive 5-year survival outcomes, similar to those in previous studies conducted by Habr-Gama [26,27,28,29,30]. However, a few studies highlighted some negative aspects of this WW approach, demonstrating slightly lower survival rates in WW patients than those documented before, especially in patients younger than 55 years [31], in addition to a significantly higher rate of distant metastasis observed among patients who had local regrowth when compared to that in those who did not [32]. These outcomes proved the necessity of properly selecting patients when opting for this non-operative path.

Today’s widespread TNT protocol was brought to light as a solution to the high distant metastasis (DM) rates witnessed in high-risk LARC patients, with the added benefit of both radiotherapy and chemotherapy, and as an alternative to the adjuvant chemotherapy option, for which low compliance rates driven by postoperative complications have been proven [33,34,35]. One of the earliest clinical trials to test the outcomes of TNT was a phase II trial conducted by Chau et al. in 2006, in which TNT tested on poor-risk rectal cancer patients resulted in major tumor regression, rapid symptomatic relief, and high rates of R0 resection after a median follow-up of 23 months [36]. Another promising role of TNT was demonstrated by an observational study carried out by Cercek et al. [37], which compared TNT to preoperative CRT followed by surgery and chemotherapy, reporting that TNT improved the pathologic complete response (pCR) in patients who underwent surgery and sustained a cCR in those who were under surveillance, which highlighted the role of TNT in enhancing WW outcomes, paving the way for future organ preservation (OP) approaches. TNT was integrated further into clinical practice around 2020, after the phase III RAPIDO trial demonstrated higher rates of compliance to systemic therapy when giving TNT to high-risk LARC patients compared to those for the standard CRT regimen, in addition to significantly higher pCR rates and a lower cumulative probability of DM. In 2023, the long-term update of the RAPIDO trial showed a higher rate of locoregional recurrence with short-course RT in patients with high-risk LARC, making long-course CRT preferrable in this specific patient population [38,39].

**Figure 1:**
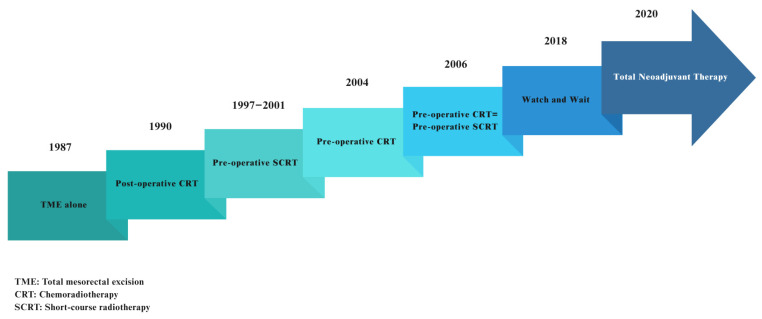
The evolution of locally advanced rectal cancer treatment options: from surgery alone to TNT.

Figure 1 summarizes the evolution of the new advances in the treatment of LARC from the 1980s to the 2020s.

In recent years, several clinical trials have been conducted to improve the standard of care for LARC, focusing on overcoming the challenges associated with the current treatment modalities and exploring alternative strategies. As a result, patients currently have access to a broader range of treatment options, increasingly tailored to their individual risk profiles and personal preferences.

## 4. Radiation Therapy for LARC Today: Innovations and Updates

### 4.1. Radiotherapy as a Component of Total Neoadjuvant Therapy in LARC

Some of the most notable trials that we will discuss here to evaluate the TNT protocol include the STELLAR, RAPIDO, PRODIGE-23, and TNTCRT randomized phase III trials (Table 1).

The STELLAR phase III trial aimed to establish non-inferiority between the preoperative TNT regimen of short-course radiotherapy followed by neoadjuvant chemotherapy and the previous standard of care of preoperative chemoradiotherapy in patients with LARC in the distal or middle third of the rectum [40]. This hypothesis arose after the Polish phase III trial demonstrated significantly better overall survival and acute toxicity rates among cT3–T4 patients who received SCRT followed by consolidation chemotherapy (only three cycles of FOLFOX4) when compared to those in CRT patients [41]. Similarly, the 3-year outcomes of the STELLAR trial showed significantly better OS rates among TNT patients with similar DFS, metastasis-free survival (MFS), and local recurrence (LR) rates when compared to those in the CRT group, establishing non-inferiority between both regimens. It is important to note, however, that the rate of acute-grade III–V toxicities during preoperative treatment was significantly higher in the TNT group.

Importantly, the involvement of positive lateral pelvic lymph nodes should be considered during the discussion of treatment planning, both as a prognostic indicator and a potential rationale for treatment intensification. A post hoc analysis of the STELLAR trial revealed that patients with lateral pelvic lymph node metastases exhibited significantly lower 3-year DFS, OS, and MFS rates compared to these rates in those without such involvement; this was observed in both the experimental and control arms [42]. As for operative considerations, it has been recommended that at least 11 LNs should be retrieved in LARC patients to guide the decisions regarding adjuvant chemotherapy better [43].

Another similar trial is the phase III RAPIDO trial. Its inclusion criteria included high-risk LARC patients, who were divided between (1) an experimental group treated using TNT as a short-course radiotherapy with 6 cycles of CAPOX or 9 cycles of FOLFOX4 followed by TME and (2) a standard-of-care group treated using long-course chemoradiotherapy (28–25 × 1.8–2.0 Gy with concurrent capecitabine) followed by TME and optional adjuvant chemotherapy given as 8 cycles of CAPOX or 12 cycles of FOLFOX4. The 3-year follow-up showed excellent results for the TNT arm, with a significantly lower 3-year disease-related treatment failure (DrTF) rate and cumulative probability of DM, a significantly higher pCR, and a similar 3-year cumulative probability of locoregional failure (LRF) and OS when compared to these values in the standard group [39]. Nevertheless, these outcomes were faced with some criticism considering that the optional adjuvant chemotherapy may have biased the results in favor of the TNT arm [44] and should have been given unanimously in the control group to account for any oncologic benefits. More importantly, the results at the 5-year follow-up demonstrated significantly higher 5-year LR rates in the TNT arm compared to those in the standard arm (10.2% vs. 6.1% with *p* = 0.027) in patients who underwent R0 or R1 resection [45].

The phase III PRODIGE-23 trial had a similar aim to the RAPIDO trial but used a different TNT sequence, comprising an intense neoadjuvant chemotherapy protocol followed by long-course chemoradiotherapy [46]. LARC patients with cT3 tumors with a risk of local recurrence or cT4 resectable tumors were randomly assigned into either the TNT group receiving neoadjuvant chemotherapy with six cycles of mFOLFIRINOX, followed by chemoradiotherapy of 50 Gy/25 fractions + capecitabine, or to the standard-of-care group receiving the same chemoradiotherapy, both followed by TME and adjuvant chemotherapy given as FOLFOX6 or capecitabine. The 3-year outcomes demonstrated significantly higher DFS, MFS, and pCR rates in the TNT group compared to those in the control, which were reproduced at the 7-year follow-up, in addition to a significantly higher OS rate [47].

Both the RAPIDO and PRODIGE-23 trials were able to achieve their primary aim and to present the necessary evidence of the ability of TNT to increase the rate of pCR and, more importantly, to reduce the rate of distant metastasis in LARC patients. However, considering the higher locoregional recurrence rate in the 5-year follow-up of the RAPIDO trial, the ASCO 2024 guidelines recommend neoadjuvant long-course CRT over short-course RT when opting for TNT in high-risk LARC patients [48].

The same ASCO meeting held in 2024 presented the TNTCRT phase III clinical trial for LARC. This trial divided high-risk LARC patients between arm A, receiving TNT as long-course chemoradiotherapy (LCRT) (given as 50–50.4 Gy in 25–28 fractions) with six cycles of neoadjuvant CAPOX followed by TME, and arm B, receiving neoadjuvant chemoradiotherapy (NCRT) followed by TME and adjuvant CAPOX. The 3-year follow-up outcomes presented significantly higher DFS, MFS, and pCR rates in the TNT arm compared to those in arm B, further showing the superiority of TNT over conventional preoperative chemoradiation, especially for high-risk LARC [49].

After establishing that TNT was the new standard for the treatment of high-risk LARC through several phase III trials, the CAO/ARO/AIO 12 phase II trial aimed to assess the preferred sequence of FOLFOX chemotherapy and radiotherapy in TNT in terms of pCR and other oncologic outcomes [50]. LARC patients were divided between group A, receiving TNT as induction chemotherapy followed by CRT, and group B, receiving it as CRT followed by consolidation chemotherapy, where CRT was administered as 50.4 Gy in 28 fractions using intensity-modulated radiotherapy (IMRT) concomitant with fluorouracil and oxaliplatin. The three-year outcomes yielded a higher pCR in group B compared to that in group A, with an odds ratio of 1.69, in addition to lower grade 3 and 4 radiation toxicity rates. The other oncologic outcomes, including the 3-year DFS, LR, and DM rates, were similar between both groups (Table 2).

### 4.2. The Role of Radiotherapy in Organ Preservation for LARC

Another emerging treatment strategy for select patients with LARC is non-operative management (NOM), also known as watch and wait (WW). It aims to spare the organs and avoid the morbidity associated with TME, including in sexual, urinary, and bowel functions [51].

For patients who achieve a complete clinical response to neoadjuvant therapy, NOM relies on substituting surgery with thorough and ongoing surveillance to enable the early detection of local regrowth and timely intervention. In 2021, an international consensus outlined a recommended watch-and-wait follow-up protocol for the first five years following TNT which includes regular assessments using serum carcinoembryonic antigen (CEA) testing, digital rectal examination (DRE), proctoscopy, pelvic MRI, and chest and/or abdominal CT imaging [52]. Table 3 summarizes the recommended schedule for each test.

The main condition to qualify for the NOM protocol is achieving complete clinical response (cCR) after neoadjuvant treatment, which raises the question, what are the best strategies to reach cCR?

The OPRA phase II trial is one of the latest clinical trials involving the WW method using TNT [53]. It tests the preferred sequence of chemotherapy and radiotherapy in TNT and the efficacy of TNT in achieving organ preservation in LARC. Patients with stage II and III rectal cancer were randomly assigned into a group receiving neoadjuvant chemotherapy before CRT called the induction chemotherapy group (INCT-CRT) and a group receiving the same chemotherapy after CRT called the consolidation chemotherapy group (CRT-CNCT), where CRT was administered via IMRT or 3DRT as 45 Gy/25 fractions to the pelvis with a boost of 50–56 Gy to the primary tumor and positive lymph nodes with concurrent capecitabine or infusional 5 FU. The clinical tumor response was divided into three categories, complete (cCR), near-complete (nCR), and incomplete (iCR), according to a three-tier schema which required evaluation through DRE, endoscopy, and MRI [54] (Table 4). Patients with a complete or a near-complete response after restaging 8 +/−4 weeks post-TNT were offered watchful waiting, while TME was performed in those with an incomplete response.

The results at the 3-year follow-up showed a similar 3-year DFS between the induction and consolidation chemotherapy groups (76%), which was in line with the 75% 3-year DFS rate observed historically in patients who underwent TME following preoperative chemoradiation. The rates of local recurrence-free survival and distant-metastasis-free survival were also similar in both groups. A more important finding was that 74% of the patients were eligible for NOM, and among those, the 3-year TME-free survival rate was significantly higher in the consolidation chemotherapy group. The five-year follow-up reached the same outcomes, with a five-year TME-free survival of 39% versus 54% in favor of the consolidation chemotherapy regimen in terms of organ preservation (Table 5) [55].

A total of 36% of patients who underwent WW had regrowth, where 94% and 99% of these incidents occurred within the first 2 and 3 years, respectively, which emphasizes the need for careful patient monitoring during this time interval. In addition, it is important to explain to patients the expected low probability of organ preservation when positive lateral lymph nodes (LLN) ≥ 4 mm persist after TNT, as demonstrated by a recent post hoc analysis of the OPRA trial [56].

A secondary analysis of the OPRA trial segregated the oncologic outcomes observed in the trial further among the three clinical tumor response grades, which included a complete, near-complete, and incomplete response [57]. It demonstrated that the 3-year probability of OP was significantly higher for patients with a cCR compared to those with an nCR, with a lower risk of local regrowth observed at 2 years in the cCR group compared to that in the nCR group (Table 6). There was also a significant difference among the three clinical tumor response grades in terms of the DFS, LRFS, DMFS, and OS. This observation suggests that the clinical tumor response grade can be regarded as a prognostic factor in terms of survival outcomes, as well as organ preservation expectations, and hence should be considered during treatment planning discussions.

The OPRA trial provided a landmark of the ability of TNT to lead the way to non-operative management and subsequent organ preservation in LARC patients, with no apparent oncologic disadvantages in patients who achieve a complete clinical response and follow a rigorous surveillance schedule.

This was reinforced by a pooled analysis comparing the OPRA trial with the CAO/ARO/AIO-12 trial [58]. Considering that all patients in the latter trial underwent TME after TNT, the similar oncologic outcomes between both trials concluded that WW is a safe and non-inferior treatment option for patients who achieve a complete response to TNT.

Another promising trial in the quest for non-operative management is the ongoing JANUS seamless phase II/III trial, which aims to provide insight into the optimal consolidation chemotherapy regimen to achieve a cCR [59]. It includes LARC patients requiring abdominoperineal resection or coloanal anastomosis with a distal margin within 12 cm of the anal verge, who will be randomized into two groups that will receive neoadjuvant LCRT followed by either consolidation doublet (mFOLFOX6 or CAPOX) or triplet chemotherapy (mFOLFIRINOX) for 3–4 months. The LCRT will be given as 45 Gy in 25 fractions in addition to a boost of 9 Gy in 5 fractions. Phase II of the trial has a primary endpoint of comparing the cCR rates between groups, while phase III aims to compare the DFS rates, hopefully filling a gap on the road to NOM.

**Figure 2:**
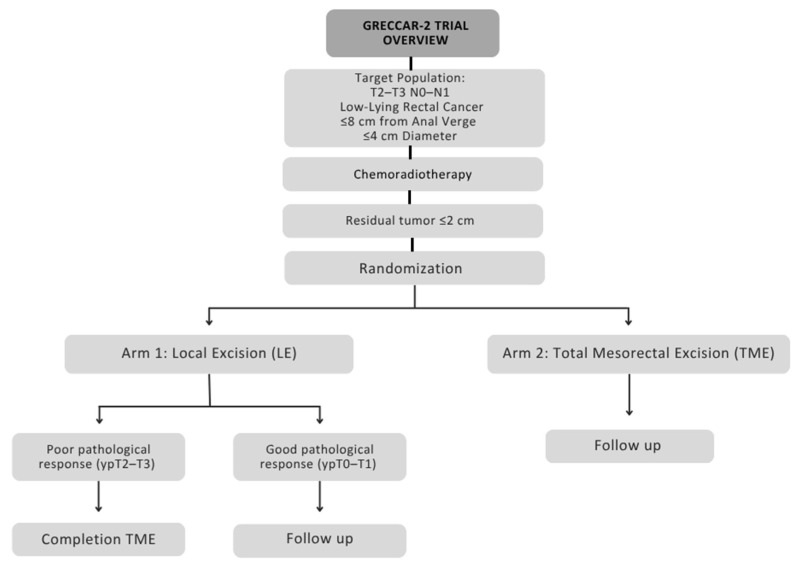
The study design of the GRECCAR-2 trial.

While most organ preservation trials have focused on TME vs. observation, some studies have offered another less invasive option, which is local excision of the tumor. Local excision spares the removal of the entire rectum and instead requires surgical transanal full-thickness rectal wall excision with a bowel margin of 1 cm, keeping most of the rectum functional. GRECCAR-2 was a multicenter phase III trial considered the first of a kind to compare local excision with TME in downstaged low rectal cancer. It included patients with cT2–T3 and N0–N1 (≤3 positive nodes ≤ 8 mm) low rectal cancer that was ≤8 cm from the anal verge, sized ≤ 4 cm, and eligible to receive chemoradiotherapy and major surgery [60]. These patients received neoadjuvant CRT given as 50 Gy in 25 fractions with concomitant fluorouracil, and those who achieved a good clinical response 8 weeks later, defined as a residual tumor of ≤2 cm, were randomized between a local excision group and a TME group. To ensure patient safety, for patients in the local excision group who had a poor pathological response (ypT2–T3 or R1), a completion total mesorectal excision was performed 1–4 weeks after local excision (Figure 2). The 5-year follow-up demonstrated similar oncologic outcomes between both groups, including LR, DM, OS, DFS, and cancer-specific mortality rates (Table 7). These outcomes suggest a new option for patients with small T2–T3 low rectal cancer that wish to avoid the morbidity associated with TME, given they respond well to CRT.

The STAR-TREC phase III trial is another prominent study exploring the feasibility of organ preservation in patients with LARC, particularly those with lower-risk rectal tumors [61]. It is expected to yield essential information regarding the feasibility of organ preservation using neoadjuvant radiotherapy alone, in addition to the preferred radiotherapy course. In this trial, 344 patients with cT1–T3b N0 tumors that were ≤40 mm in diameter were enrolled and divided between a TME arm and an organ preservation arm according to their preference. The patients who chose organ preservation were further randomized between short-course radiotherapy and a longer course of chemoradiotherapy. Patients who achieve a cCR will be eligible for WW surveillance while those with an incomplete response will be recommended for local excision or TME depending on a pathologic assessment. The primary outcome will be the rate of organ preservation at 30 months, while the secondary outcomes will mainly include treatment-related toxicities, 3-year LC and DFS rates, and 5-year OS rates. The preliminary results at 1 year were recently announced at ESTRO 2025 [62], indicating that 80% of the patients in the long-course chemoradiotherapy arm and 61% of those in the short-course radiotherapy arm had TME-free survival, with minimal side effects reported. This outcome may mark the introduction of a major shift in the LARC guidelines, which ought to encourage non-surgical approaches.

### 4.3. Optimizing Organ Preservation Through Radiotherapy Dose Escalation in LARC

**Figure 3:**
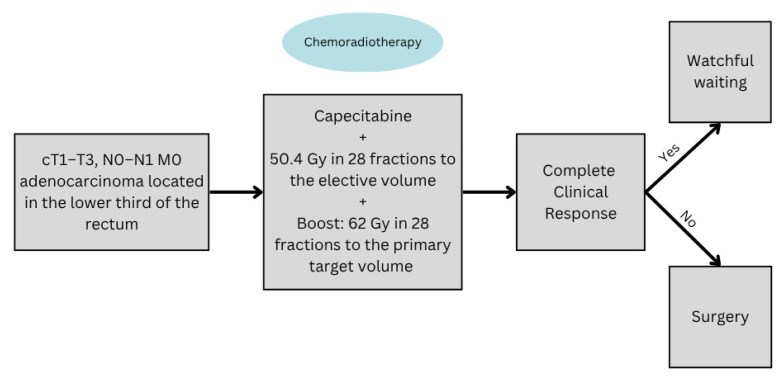
The study design of the Danish watchful waiting phase II trial.

A Danish multicenter phase II trial was one of the most notable studies to report on radiation dose escalation as an organ preservation strategy [63]. It included patients with primary resectable T1–T3, N0–N1, M0 low rectal adenocarcinoma, where the N1 nodes had to be at the level of the tumor and included in the primary tumor volume. Radiotherapy was given concomitantly with capecitabine as IMRT and consisted of 50.4 Gy in 28 fractions to the elective volume, which included the mesorectal and presacral lymph nodes, along with the lateral pelvic lymph nodes, in addition to a concomitant boost of 62 Gy in 28 fractions to the primary target volume, which included the primary tumor and the rectal circumference at its level. Patients who achieved a cCR 6–12 weeks post-CRT were eligible for watchful waiting, while patients with an incomplete clinical response or regrowth were planned for surgery (Figure 3). The results were encouraging, with 86% of patients achieving a cCR after CRT alone, and 58.9% of the patients sustaining locoregional tumor control after 2-years of follow-up. The MFS and OS at 30 months were 85.4% and 94.8%, respectively, with patients reporting acceptable rates of side effects. The analysis of the treatment planning in this trial also showed that target coverage was reached in most plans with no substantial high-dose volumes reaching the organs at risk, most importantly the bladder and the intestines [64].

Radiotherapy dose escalation for LARC organ preservation was also evaluated in the phase III OPERA trial, which tested the effect of an intracavitary contact X-ray brachytherapy boost on improving the 3-year organ preservation rate, with emphasis on the toxicity profile witnessed in the WW group [65]. It included patients with an operable adenocarcinoma in the mid–low rectum (≤10 cm from the anal verge), staged as cT2–cT3a/b, smaller than 5 cm in diameter, involving less than half the rectal circumference, and with cN0–cN1 < 8 mm. These patients received neoadjuvant CRT as 45 Gy external beam radiotherapy in 25 fractions over 5 weeks with concurrent oral capecitabine, before being randomly assigned between group A receiving an external beam radiotherapy (EBRT) boost as 9 Gy in 5 fractions and group B receiving a contact X-ray brachytherapy (CXB) boost as 90 Gy in 3 fractions. Exceptionally, tumors smaller than 3 cm in group B received the CXB boost before neoadjuvant CRT, as a previous study had shown an advantage of upfront CXB radiotherapy in these tumors in terms of the clinical CR [66]. Patients with an incomplete response were scheduled for TME, while those with a cCR or an nCR were recommended for local excision or TME depending on the pathologic tumor characteristics.

The results showed a significantly higher 3-year organ preservation (OP) rate in the CXB arm, with a much larger difference when dealing with tumors smaller than 3 cm compared to tumors ≥ 3 cm. As for the toxicity-related results, early-grade 2–3 toxicity reports were similar between the two arms, while late-grade 1–2 rectal bleeding was more frequent in the CXB arm, with resolution reported three years later. Poor bowel function was assessed through a Low Anterior Resection Syndrome (LARS) score ≥ 30 and showed insignificant differences between the two arms (Table 8).

The trial is still ongoing, and more accurate results are expected after a longer follow-up period, but the current results, along with those of the Danish trial, lay a foundation for a new approach in LARC treatment which focuses on dose escalation, whether through IMRT or contact X-ray brachytherapy, as a successful technique to increase OP, especially in low-risk LARC patients with relatively smaller lower rectal tumors.

**Figure 4:**
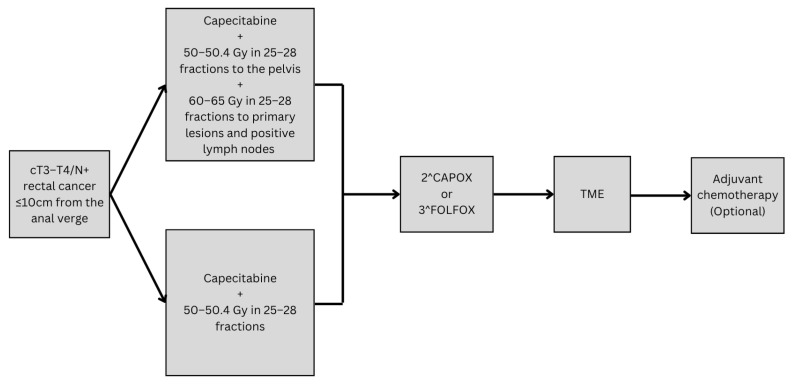
The study design of Wang et al.’s phase III trial.

A trial led by Wang et al. focused on the role of radiation dose escalation on the postoperative outcomes in LARC using magnetic-resonance-guided adaptive radiotherapy (MRgART), which is a radiotherapy technique that depends on continuously adjusting the radiation dosage to account for any changes in the tumor or the surrounding organs. It is an ongoing randomized phase III trial based in China that is testing the effectiveness of administering a simultaneous integrated boost (SIB) to both the primary lesions and positive lymph nodes using MRgART [67]. LARC patients with lower or mid tumors (≤10 cm from the anal verge) will be divided between a SIB group, receiving 60–65 Gy in 25–28 fractions to the primary lesions and positive lymph nodes and 50–50.4 Gy in 25–28 fractions to the pelvis, and a standard group receiving IMRT as 50–50.4 Gy in 25–28 fractions (Figure 4). Both groups will subsequently be treated using chemotherapy, TME, and optional adjuvant chemotherapy. The primary endpoints will be the pathological complete response rate and surgical difficulty.

### 4.4. The Selective Omission of Radiotherapy in LARC

On the other side of the spectrum is treatment de-escalation with the selective omission of radiation therapy, especially in low-risk patients, mainly to decrease toxicity-related adverse events caused by radiotherapy, including but not limited to bowel, bladder, and sexual dysfunction; an elevated risk of pelvic fractures and secondary malignancies; a reduced bone marrow reserve; infertility; and early menopause. The FOWARC phase III trial was designed to test the safety of omitting preoperative RT in patients with stage II/III rectal cancer. This trial randomly assigned LARC patients into a fluorouracil plus radiotherapy group, a mFOLFOX6 plus radiotherapy group, or a mFOLFOX6 alone group, where radiotherapy was administered as 46–50.4 Gy in 23–25 fractions, and all groups were subsequently treated with surgery and adjuvant chemotherapy [68]. Both the 3-year and 10-year results showed similar DFS, LR, and OS rates among all groups, which supports the strategy of using neoadjuvant chemotherapy with modified FOLFOX alone for select LARC patients [68,69] (Table 9).

Table 10 summarizes some of the major studies that have described the process and outcomes of omitting radiotherapy from the treatment regimen of LARC.

The PROSPECT seamless phase II/III trial also aimed to test the safety of omitting CRT in selected patients [70]. Patients with cT2N+, cT3N−/+ rectal cancer with no more than four perirectal lymph nodes that were >10 mm, in whom the CRM was not threatened, and who were candidates for sphincter-sparing surgical resection were randomly divided between two groups, a control group, which comprised CRT given as 50.4 Gy in 28 fractions with either capecitabine or 5FU followed by TME and optional adjuvant chemotherapy, and an intervention group, which comprised six cycles of mFOLFOX6 followed by the response-guided use of CRT and then TME and optional adjuvant chemotherapy, where a tumor regression rate of >20% excluded CRT use. The 5-year follow-up demonstrated similar DFS, local recurrence-free survival, OS, and pCR rates between both groups, with 90% of the patients in the intervention group achieving > 20% tumor regression and skipping CRT. Moreover, the intervention group experienced a significantly higher R0 resection rate (98.9% vs. 91.7%, *p* = 0.094), with satisfactory patient-reported outcomes [71], including lower diarrhea, fatigue, and neuropathy rates, and better bowel and sexual function 12 months after surgery.

These outcomes proved the non-inferiority of neoadjuvant chemotherapy alone to neoadjuvant CRT in low-risk mid–upper rectal cancer, which means radiotherapy can be safely omitted in selected patients to reduce toxicity-related morbidity without jeopardizing survival outcomes.

The similar CONVERT phase III trial failed to prove the non-inferiority of neoadjuvant CRT to neoadjuvant CAPOX alone in terms of the locoregional recurrence-free survival; however, it did show similar oncologic outcomes, including 3-year DFS, OS, and pCR rates, between both groups, in addition to significantly lower perioperative distant metastases and grade 2 long-term toxicity rates in the chemotherapy group [72,73]. Hence, this trial could still support the safety of radiotherapy omission in selected LARC patients.

A prospective observational study called the OCUM study tested the possibility of omitting radiotherapy by offering upfront surgery instead of chemotherapy [74]. This study included stage cT2–T4 rectal cancer patients who had any cN and cM0 and were undergoing elective surgery with curative intent (R0, R1). These patients were divided between two groups based on their MRI findings: (1) an upfront surgery group which only included cT2 and cT3 patients with no involved or threatened mesorectal fascia and (2) a group of high-risk patients whose MRIs showed an involved or threatened MRF (a distance between the tumor and the MRF ≤ 1 mm) and cT4 or cT3 carcinomas of the lower rectal third, who received neoadjuvant CRT before TME, given as 50.4 Gy/1.8 Gy with concurrent fluorouracil. The 3-year follow-up showed similar LR rates between both groups, noting that around 27% of the patients who underwent primary surgery turned out to have pathological stage I cancer. These outcomes also suggest the possibility of omitting radiotherapy in low-risk LARC patients given that both the MRI diagnosis and surgery are performed with gold-standard qualities.

Previous studies proving MRI’s predictive role for surgical outcomes in LARC were the basis of many studies like OCUM but were not as highly regarded due to their limited sample sizes. One important study is called MERCURY, which was an observational prospective study that proved how high-resolution MRI could accurately predict a clear CRM, which is, in turn, the main predictor of local recurrence [75]. This offered great help for future trials investigating the safety and feasibility of omitting radiotherapy in LARC patients, as patients with an MRI-proven distance of ≤1 mm between the tumor and the MRF were considered to have a high risk of a positive CRM and ought to subsequently be recommended for preoperative treatment. The specificity of high-resolution MRI in predicting a negative CRM in the MERCURY study was 92%.

Later on, the QUICKSILVER phase II trial tested the effectiveness of MRI as a prognostic tool that could aid in the selection of patients eligible for primary surgery [76]. Patients who were considered to have a “good prognosis” based on several MRI findings, including MRF involvement, cT stage, and EMVI, were deemed eligible for primary surgery, and the results showed that only 4.9% of these patients had a positive circumferential resection margin, which was considered an acceptable rate.

The only clinical trial which has investigated the long-term survival of LARC patients who have undergone primary surgery based on an MRI-proven negative CRM is the PSSR trial. This randomized phase III trial included LARC patients with middle rectal tumors who had a negative MRI-proven CRM and divided them between an experimental group who underwent primary TME and a control group who underwent CRT followed by TME and adjuvant chemotherapy [77]. There were significantly more 3-year DFS events in the experimental group than the control group, and despite the difference in the DFS rates between the two groups being within the non-inferiority margin, this study was terminated, as the risk of recurrence was not acceptable according to clinical practice. Unlike the OCCUM study, the PSSR trial demonstrated a high risk of recurrence in the upfront surgery group, which could be attributed to the fact that high-risk patients were included in the trial. This outcome is proof that only selected patients, specifically low-risk LARC patients, are eligible for the omission of radiotherapy as a neoadjuvant treatment.

### 4.5. Combining Radiotherapy with Immunotherapy

As opposed to its counterpart, MSS LARC has not standardly been known for its sensitivity to immunotherapy. However, more recent trials have explored the efficacy of short-course radiotherapy when combined with immunotherapy, demonstrating promising results. The recent single-arm phase II NeoCaCRT trial included patients with pMMR/MSS non-metastatic LARC located below the peritoneal reflection, who received SCRT followed by six cycles of cadonilimab and mFOLFOX6. The preliminary results at 9.7 months presented a satisfactory pCR of 37% and a major pathologic response (MPR) of 55.6% post-TME or local excision, with an acceptable safety profile, supporting further evaluation in a phase III trial [78]. Similarly, the phase II Averectal study, which employed the same treatment protocol while using the PD-L1 antibody avelumab as the immunotherapy regimen, resulted in a pCR of 37.5% and an MPR of 67.5% at 3 years, with low local recurrence and toxicity rates [79]. These results are coherent with the conclusion of the systematic review led by Yang et al., which demonstrated a pooled pCR of 38% and an MPR of 60% in its subgroup analysis of pMMR/MSS patients while suggesting a preference for short-course radiotherapy as compared to long-course RT (a pCR of 51% vs. 30%, respectively) and a sequential immuno-chemoradiotherapy regimen as opposed to a concurrent regimen (a pCR of 40% vs. 30%, respectively) [80]. This approach represents a promising new treatment strategy for patients with MSS LARC. Further studies are needed to identify the most responsive patients to this route, in addition to the optimal radiotherapy and immunotherapy regimens to pair together.

### 4.6. The Role of Adjuvant Radiotherapy in LARC

The use of adjuvant radiotherapy for LARC has significantly decreased since the early 2000s after several reputable trials proved the superiority of neoadjuvant treatment over the former [14,15,18]. However, it is still being considered in select patients, such as patients with cT1–T2 N0 tumors who are upstaged postoperatively (pT3/T4, node-positive, positive margins). Upstaging of rectal tumors has been estimated to occur in 16% to 25% of cT1–T2N0 patients [81]. The benefit of adjuvant radiation therapy has been reported in several retrospective studies, mainly in patients with positive surgical margins and N2 disease [82,83,84,85]. Nevertheless, contemporary trials testing the benefit of adjuvant radiotherapy in the era of modern effective systemic therapies are needed since adjuvant radiotherapy is associated with significant bowel toxicities [86,87].

### 4.7. The Role of Intraoperative Radiotherapy (IORT) in LARC

Intraoperative radiotherapy (IORT) involves delivering a single high dose of radiation directly to the tumor bed while the area is exposed during surgery, which allows for the eradication of residual cells after the tumor is resected. Radiation can be delivered using electron beams, low- or high-energy X-rays, and high-dose-rate (HDR) brachytherapy [88], each having a unique set of advantages and disadvantages. Similar to adjuvant radiotherapy, IORT is not part of the standard treatment in LARC, but it does play a complementary role in select cases. Several studies have demonstrated the oncologic and survival benefits of IORT in primary and recurrent LARC patients who have undergone tumor resection with local residual disease or close margins [89]. The current data suggest the importance of treating residual microscopic or gross disease with a radiation dose ≥ 60 Gy [89,90], which poses a higher risk of toxicity to the surrounding tissues in the pelvis. The rationale behind IORT’s advantage lies behind the small volume of normal tissue included in the field of IORT owing to the ability to mobilize the surrounding tissues during surgery, allowing a single dose of 10 to 20 Gy to be delivered in addition to the standard preoperative dose of conventionally fractionated 45–50 Gy [89].

Haddock et al.’s review reported excellent local control rates in patients with primary LARC who underwent IORT, ranging between 84 and 100% for R0 and R1 resections [89]. Patients with R2 resections also seemed to benefit from IORT, with the local control rates varying between 57 and 73%, superior to the historical outcomes. Liu et al. reported similar outcomes in their systematic review, which demonstrated significantly improved 5-year local control rates without a survival benefit among rectal cancer patients who underwent IORT when compared to these rates in those who did not [91].

Currently, several guidelines recommend IORT, if available, in patients with very close or positive margins after resection only as an additional boost post-standard neo-adjuvant CRT, with special consideration for patients with T4 disease after TNT and patients with recurrent cancers [8,92,93]. The 2020 ESTRO/ACROP guidelines also specified the recommended dosing for IORT depending on margin status, where 10 to 12.5 Gy was considered best for R0 resection, 12.5 to 15 Gy best for R1 resection, and 15 to 20 Gy best for R2 resection [93].

## 5. Discussion

Over the past few years, the management of locally advanced rectal cancer has evolved significantly, with recent guidelines increasingly embracing patient-centered approaches that prioritize not only oncologic efficacy but also minimizing toxicities and maintaining patients’ quality of life.

Choosing the optimal treatment plan begins by assessing the patient’s rectal tumor MMR status, as patients with dMMR have a great response to immunotherapy and chemoradiotherapy and surgery can potentially both be omitted. In contrast, patients with pMMR tumors require a more individualized treatment approach, involving a comprehensive evaluation of clinical risk factors, comorbidities, and tumor location and stage, as well as patient preferences and expectations.

The role of radiotherapy in LARC treatment remains widely diverse, specifically in low-risk patients, where on the one hand, there is a proven advantage of radiation dose escalation in enhancing the complete response rates and subsequent organ preservation and bypassing surgery-related morbidities, while on the other hand, there is a possibility of omitting RT altogether in upper rectal tumors and selected patients eligible for sphincter-sparing surgery who achieve > 20% tumor regression upon neoadjuvant chemotherapy, sparing the risk of radiation toxicity. Here lies the importance of patient-related factors and individual preferences in guiding treatment decisions. For instance, in a patient with a history of prior pelvic radiation therapy or a young premenopausal woman, the “no RT”/surgical approach may be favored to avoid the potential toxicities of pelvic radiation, whereas a patient with a very low rectal tumor requiring an abdominoperineal resection or an elderly patient with medical comorbidities may be treated better with a “no surgery”/dose-escalated RT strategy. These scenarios highlight the significance of a comprehensive and transparent discussion—not only within a multidisciplinary tumor board but also directly with the patient—in order to present all oncologically sound treatment options and their respective side effect profiles and to align the chosen strategy with the patient’s lifestyle, values, and personal preferences.

High-risk patients are advised to opt for TNT, which consists of administering both radiotherapy and chemotherapy before surgery, as its significant role in improving pathological and clinical complete response rates, in addition to reducing distant metastasis and improving survival, has been proven several times, earning it its position in the current NCCN guidelines. To date, there is no clear consensus on the optimal sequencing of radiotherapy and chemotherapy in TNT for LARC. A rational approach would be to stratify eligible patients based on their risk of distant metastasis versus local recurrence. For patients at high risk of systemic spread—such as those with cT4 tumors, EMVI, or involved lateral pelvic lymph nodes—an induction chemotherapy-first strategy, particularly with intensified regimens like mFOLFIRINOX, as used in the PRODIGE-23 trial, may be advantageous. On the other hand, for patients with tumor features suggesting a higher risk of local recurrence, such as a threatened CRM, for example, initiating treatment with chemoradiotherapy—as in the CAO/ARO/AIO 12 phase II trial—is a reasonable strategy. The CAO/AIO/ARO-12 trial proved higher pCR rates when administering this sequence of neoadjuvant treatment.

It is worth noting that some patients with high-risk features might be interested in organ preservation or may be medically inoperable, in which case the abovementioned TNT approach is followed with watchful waiting if a complete clinical response is achieved. The OPRA trial demonstrated that long-course radiotherapy followed by consolidation chemotherapy is highly effective in enhancing the TME-free survival and thus organ preservation, making it an attractive option for LARC patients considering NOM. For patients seeking organ preservation, radiation dose escalation should be considered, if the dose constraints to organs at risk are respected. This is preferably carried out using advanced techniques such as IMRT or the addition of a brachytherapy boost. When following the non-operative approach, it is important to highlight the significance of following a rigorous surveillance schedule, especially in the first 3–5 years after treatment.

To optimize the outcomes for high-risk patients with LARC further, an interesting strategy would be to combine the benefits of intensive chemotherapy (mFOLFIRINOX), to decrease the risk of distant metastasis, with the sequencing of CRT first followed by chemotherapy second. The results of the ongoing JANUS trial, randomizing patients to receive CRT followed by FOLFOX versus CRT followed by FOLFIRINOX, are awaited to test the efficacy of this strategy.

**Figure 5:**
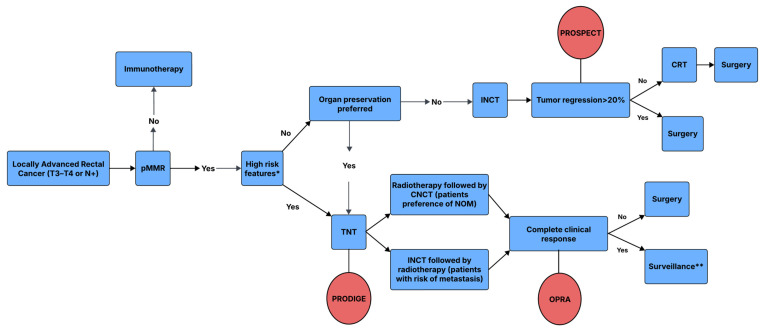
Treatment algorithm for locally advanced rectal cancer. CRT: chemoradiotherapy; CNCT: consolidation chemotherapy; INCT: induction chemotherapy; TNT: total neoadjuvant therapy. * High-risk features include cT4a/b, cN2, enlarged lateral lymph nodes considered to be metastatic, extramural vascular invasion (EMVI+), and involved mesorectal fascia (MRF+), which are evident on MRI. ** Surveillance is an option based on shared decision-making between the patient and the physician, and clinical judgment should be employed to assess a patient’s ability to comply with the surveillance protocol.

Figure 5 shows a simplified treatment algorithm for LARC after the most recent updates.

## 6. Conclusions

The treatment paradigm for locally advanced rectal cancer has significantly evolved from surgery alone to a multimodal approach that can involve chemotherapy, radiotherapy, and surgery. This review summarizes the most recent updates in the different treatment protocols offered to LARC patients with MSS tumors, focusing on the role of radiotherapy.

Multiple oncologically sound treatment pathways can now be considered. We emphasize that patient preferences, as well as patient- and tumor-specific factors, significantly influence the selection of an appropriate treatment strategy. Thus, there is a need for open, multidisciplinary discussions—not only within the tumor board but also in direct consultation with the patient—to present all the options, to discuss their potential benefits and side effects, and to ensure that the chosen pathway aligns with the patient’s preferences and treatment goals.

## Figures and Tables

**Table 1:** Overview of randomized trials involving total neoadjuvant therapy for locally advanced rectal cancer.

Trial	STELLAR	RAPIDO	PRODIGE-23	TNTCRT
Eligibility	cT3 or cT4 and/or N+, M0, tumor located in the distal or middle third of the rectum	cT4a,b, extramural invasion, cN2 disease, enlarged lateral lymph nodes, or involved mesorectal fascia	cT3 with a risk of local recurrence or cT4 resectable rectal cancer with no metastasis	cT4a,b that is resectable, cT3c,d with extramural venous invasion, cN2, involved mesorectal fascia, or enlarged lateral lymph nodes
Experimental group	SCRT → 4^CAPOX → TME → 2^CAPOX	SCRT → 6^CAPOX or 9^FOLFOX → TME	6^FOLFIRINOX → CRT → TME → 6^FOLFOX6 or 4 capecitabine	LCRT → 6^CAPOX → TME
Control group	LCRT → TME → 6^CAPOX	LCRT → TME → +/− 8^CAPOX or 12^FOLFOX4	LCRT → TME → 12^FOLFOX6 or 8^ capecitabine	LCRT → TME → adjuvant CAPOX
Primary outcome	Disease-free survival	Disease-related treatment failure	Disease-free survival	Disease-free survival
Median follow-up	2.9 years	5.6 years	6.9 years	3.7 years
Outcomes at 3 years (experimental vs. control)				
DFS %	64.5 vs. 62.3 *		76 vs. 69 *	77 vs. 67.9 *
DrTF %		23.7 vs. 30.4 *		
OS %	86.5 vs. 75.1 *	89.1 vs. 88.8	91 vs. 88	90.3 vs. 87.9
LRF %	8.4 vs. 11	8.3 vs. 6	4 vs. 6	
MFS %	77.1 vs. 75.3	20 vs. 26.8 *^a^	79 vs. 72 *	83 vs. 74.2 *
pCR %	21.8 vs. 12.3 *	28 vs. 14 *	28 vs. 12 *	27.5 vs. 9.9 *
Outcomes at 5 years (experimental vs. control)				
DrTF %		27.8 vs. 34 *		
OS %		81.7 vs. 80.2		
LRF %		11.7 vs. 8.1 (10.2 vs. 6.1 *†)		
DM %		23 vs. 30.4 *		
Outcomes at 7 years (Experimental vs. Control)				
DFS %			67.6 vs. 62.5 *	
OS %			81.9 vs. 76.1 *	
LRF %			5.3 vs. 8.1	
MFS %			79.2 vs. 72.3 *	

Abbreviations: DFS: disease-free survival; DrTF: disease-related treatment failure; DM: distant metastasis; LCRT: long-course chemoradiotherapy; LRF: locoregional failure; MFS: metastasis-free survival; OS: overall survival; pCR: pathologic complete response; SCRT: short-course radiotherapy; TME: total mesorectal excision. * Statistically significant (*p*-value < 0.05). ^a^ Distant metastasis (DM) rate. † LRF in patients who underwent R0 or R1 resection.

**Table 2:** An overview of the CAO/ARO/AIO-12 phase II trial.

Trial	CAO/ARO/AIO-12
Eligibility	cT3–T4 and/or N+ in the middle or lower third of the rectum, where cT3 in the middle third should be >cT3b
Group A (induction chemotherapy)	3^FOLFOX → CRT → TME
Group B (consolidation chemotherapy)	CRT → 3^FOLFOX → TME
Primary outcome	Pathologic complete response
Median follow-up	3.6 years
Outcomes at 3 years (induction vs. consolidation)	
pCR %	17 vs. 25 *
DFS %	73 vs. 73 (*p*-value = 0.82)
OS %	92 vs. 92 (*p*-value = 0.81)
LRR %	6 vs. 5 (*p*-value = 0.67)
DM %	18 vs. 16 (*p*-value = 0.52)

Abbreviations: CRT: chemoradiotherapy; DFS: disease-free survival; DM: distant metastasis; LRR: locoregional recurrence; OS: overall survival; pCR: pathologic complete response; TME: total mesorectal excision. * Group B (*p*-value < 0.001), but not group A (*p*-value = 0.210), fulfilled the predefined statistical hypothesis regarding an increase in pCR.

**Table 3:** Follow-up intervals after organ preservation protocol [52].

	Year 1	Year 2	Year 3	Year 4	Year 5
CEA	q3 months	q3 months	q3 months	q6 months	q6 months
DRE, endoscopy, and pelvic MRI	q3–4 months	q3–4 months	q6 months	q6 months	q6 months
Chest and/or abdominal CT	q6–12 months	q1 year	q1 year	q1 year	q1 year

Abbreviations: CEA: carcinoembryonic antigen; DRE: digital rectal exam.

**Table 4:** The Memorial Sloan Kettering Tumor Regression Schema.

	Complete Response	Near-Complete Response	Incomplete Response
Endoscopy	Flat, white scar Telangiectasia No ulcer No nodularity	Irregular mucosa Small mucosal nodules or minor mucosal abnormality Superficial ulceration Mild persisting erythema of the scar	Visible tumor
Digital Rectal Exam	Normal	Smooth induration or minor mucosal abnormalities	Palpable tumor nodules
MRI-T2W	Only a dark T2 signal, no intermediate T2 signal AND No visible lymph nodes	Mostly a dark T2 signal, some remaining intermediate signal AND/OR Partial regression of the lymph nodes	More intermediate than a dark T2 signal, no T2 scar AND/OR No regression of the lymph nodes
MRI-DW	No visible tumor on B800–B1000 signal AND/OR Lack of a signal or a low signal in ADC map A uniform, linear signal in the wall above the tumor is okay	Significant regression of the signal on B800–B1000 AND/OR Minimal or low residual signal in ADC map	Insignificant regression of signal on B800–B1000 AND/OR Obvious low signal in ADC map

**Table 5:** An overview of the OPRA phase II trial.

Trial	OPRA
Eligibility	cT3–T4 and/or cN+
Group A (induction chemotherapy)	8^FOLFOX or 5^CAPEOX → CRT → response-guided TME or WW
Group B (consolidation chemotherapy)	CRT → 8^FOLFOX or 5^CAPEOX → response-guided TME or WW
Primary outcome	Disease-free survival
Median follow-up	5.1 years
Outcomes at 3 years (induction vs. consolidation)	
DFS %	76 vs. 76
TME-free survival %	47 vs. 60 *
LRFS %	94 vs. 94
DMFS %	84 vs. 82
Outcomes at 5 years (induction vs. consolidation)	
DFS %	71 vs. 69
TME-free survival %	39 vs. 54 *
OS %	88 vs. 85
LRFS %	94 vs. 90
DMFS %	80 vs. 78

Abbreviations: CRT: chemoradiotherapy; DFS: disease-free survival; DMFS: distant-metastasis-free survival; LRFS: local recurrence-free survival; OS: overall survival; TME: total mesorectal excision; WW: watch and wait. * Statistically significant (*p*-value < 0.05).

**Table 6:** The 3-year outcomes of the secondary analysis of the OPRA trial.

	CCR	NCR	ICR	
Outcomes at 3 Years				
OP %	77	40	-	*p*-value < 0.01
DFS %	88	69	56	*p*-value < 0.001

Abbreviations: CCR: complete clinical response; DFS: disease-free survival; ICR: incomplete clinical response; NCR: near-complete response; OP: organ preservation.

**Table 7:** The 5-year outcomes of the GRECCAR-2 trial.

	LE Group	TME Group
Outcomes at 5 Years		
LR %	7	7 *
DM %	18	19 *
OS %	84	82 *
DFS %	70	72 *
Cancer-specific mortality %	7	10 *

Abbreviations: DFS: disease-free survival; DM: distant metastasis; LE: local excision; LR: local recurrence; OS: overall survival; TME: total mesorectal excision. * Statistically insignificant (*p*-value > 0.05).

**Table 8:** Summary of OPERA phase III trial.

OPERA Trial	Group A (External Beam Radiotherapy Group)	Group B (Contact X-Ray Brachytherapy Group)
	CRT → EBRT Boost → Response-Guided TME or LE	(CRT → CXB Boost → Response-Guided TME or LE) †
Outcomes at 3 years		
Overall OP %	59	81 *
Tumors < 3 cm OP %	63	97 *
Tumors ≥ 3 cm OP %	55	68
Early toxicity grade 2–3%	36	44
Late toxicity grade 1–2%	11.6	62.7 *

Abbreviations: CRT: chemoradiotherapy; CXB: contact X-ray brachytherapy; EBRT: external beam radiotherapy; LE: local excision; OP: organ preservation; TME: total mesorectal excision. † Tumors smaller than 3 cm received a contact X-ray boost before CRT. * Statistically significant (*p*-value < 0.05).

**Table 9:** An overview of the FOWARC phase III trial design and results.

Trial	FOWARC
Eligibility	Stage II/III
Group 1	5^fluorouracil → radiotherapy → TME → 7^fluorouracil
Group 2	5^mFOLFOX6 → radiotherapy → TME → 6–8^mFOLFOX6
Group 3	4–6^mFOLFOX6 → TME → 6–8^mFOLFOX6
Primary outcome	Disease-free survival
Median follow-up	10 years
Outcomes at 3 years (group 1 vs. 2 vs. 3)	
DFS %	72.9 vs. 77.2 vs. 73.5 *
LR %	8.0 vs. 7.0 vs. 8.3 *
OS %	91.3 vs. 89.1 vs. 90.7 *
Outcomes at 5 years (group 1 vs. 2 vs. 3)	
DFS %	52.5 vs. 62.6 vs. 60.5 *
LR %	10.8 vs. 8.0 vs. 9.6 *
OS %	65.9 vs. 72.3 vs. 73.4 *

Abbreviations: DFS: disease-free survival; LR: local recurrence; OS: overall survival; TME: total mesorectal excision. * Statistically insignificant (*p*-value > 0.05).

**Table 10:** An overview of randomized trials and observational studies testing the omission of radiotherapy when treating locally advanced rectal cancer.

	PROSPECT	CONVERT	OCUM	PSSR
Eligibility	cT2N+, cT3N−/+ rectal cancer, ≤4 enlarged lymph nodes, no threatened CRM	cT3–T4a and/or N+ rectal cancer with no MRF involvement	cT2–T4, any cN, cM0, rectal cancer undergoing elective surgery with curative intent (R0, R1)	cT3–T4 and/or N+, tumor 6 to 12 cm from the anal verge, and MRI-proven MRF > 1 mm
Experimental group	6^FOLFOX → response-guided CRT → TME → +/− 6^FOLFOX or CAPOX	4^CAPOX → TME → 4^CAPOX	(Primary TME) ^a^	(Primary TME) ^c^
Control group	CRT → TME → +/− 8^FOLFOX or CAPOX	CRT → TME → 6^CAPOX	(CRT → TME) ^b^	CRT → TME → 5^capecitabine
Primary outcome	Disease-free survival	Locoregional recurrence free survival	Local recurrence	Disease-free survival
Median follow-up	4.8 years	4 years	5 years	2.9 years
Outcomes at 3 years (experimental vs. control)				
LRFS %		97.4 vs. 96.3		
OS %		94.1 vs. 95		
DFS %		89.2 vs. 87.9		81.82 vs. 85.37 (20 vs. 11.11 *) ‡
LR %			2.2 vs. 4.3 * (3.6 vs. 4.2) †	4.29 vs. 0 *
DM %			12.5 vs. 23.7 *	12.14 vs. 10.37
pCRM negative %			97.9 vs. 91.5 *	
pCR		11.0 vs 13.8		
Outcomes at 5 years (experimental vs. control)				
DFS %	80.8 vs. 78.6			
OS %	89.5 vs. 90.2			
LRFS %	98.2 vs. 98.4			
pCR %	21.9 vs. 24.3			

Abbreviations: CRT: chemoradiotherapy; DFS: disease-free survival; DM: distant metastasis; LR: local recurrence; LRFS: local recurrence-free survival; MRF: mesorectal fascia; OS: overall survival; pCR: pathologic complete response; pCRM: pathological circumferential resection margin; TME: total mesorectal excision. ^a^ Eligible patients include those with a tumor in the upper third of the rectum or in the middle third, that is, cT2–T3, with no involvement of the mesorectal fascia (a distance > 1 mm). ^b^ Eligible patients include those with a tumor in the lower third of the rectum or in the middle third, that is, cT4, or with the mesorectal fascia involved. ^c^ Patients also received postoperative CRT if the pathologic circumferential resection margin was positive, and adjuvant chemotherapy was administered based on the pathological stage and risk factors, as per the NCCN guidelines. * Statistically significant (*p*-value < 0.05). † Patients with middle or lower rectal cancer. ‡ DFS events.

## References

[1] World Health Organization Colorectal Cancer. https://www.who.int/news-room/fact-sheets/detail/colorectal-cancer?gad_source=1&gclid=CjwKCAjwxLKxBhA7EiwAXO0R0FH2aaMwGT9xFaeRB5d3wdNStTPuBpeJmaTLRV5kkKTDNvRh-E7ZvBoC2KUQAvD_BwE.

[2] Fazeli M.S., Keramati M.R. (2015). Rectal cancer: A review. Med. J. Islam. Repub. Iran.

[3] Siegel R.L., Giaquinto A.N., Jemal A. (2024). Cancer statistics. A Cancer J. Clin..

[4] American Cancer Society Key Statistics for Colorectal Cancer. https://www.cancer.org/cancer/types/colon-rectal-cancer/about/key-statistics.html.

[5] Cercek A., Lumish M., Sinopoli J., Weiss J., Shia J., Lamendola-Essel M., El Dika I.H., Segal N., Shcherba M., Sugarman R. (2022). PD-1 Blockade in Mismatch Repair-Deficient, Locally Advanced Rectal Cancer. N. Engl. J. Med..

[6] Yu J.H., Liao L.E., Xiao B.Y., Zhang X., Wu A.W., Cheng Y., Tang J.H., Jiang W., Kong L.H., Han K. (2024). Long-Term Outcomes of dMMR/MSI-H Rectal Cancer Treated with Anti-PD-1-Based Immunotherapy as Cu-rative-Intent Treatment. J. Natl. Compr. Canc. Netw..

[7] Hofheinz R.-D., Fokas E., Benhaim L., Price T., Arnold D., Beets-Tan R., Guren M., Hospers G., Lonardi S., Nagtegaal I. (2025). Localised rectal cancer: ESMO Clinical Practice Guideline for diagnosis, treatment and follow-up. Ann. Oncol..

[8] National Comprehensive Cancer Network (2025). NCCN Clinical Practice Guidelines in Oncology: Rectal Cancer, Version 2.

[9] Fisher B., Wolmark N., Rockette H., Redmond C., Deutsch M., Wickerham D.L., Fisher E.R., Caplan R., Jones J., Lerner H. (1988). Postoperative Adjuvant Chemotherapy or Radiation Therapy for Rectal Cancer: Results From NSABP Protocol R-011. JNCI J. Natl. Cancer Inst..

[10] Thomas P.R., Lindblad A.S. (1988). Adjuvant postoperative radiotherapy and chemotherapy in rectal carcinoma: A review of the gastrointestinal tumor study group experience. Radiother. Oncol..

[11] Medical Research Council Rectal Cancer Working Party (1996). Randomised trial of surgery alone versus radiotherapy followed by surgery for potentially operable locally advanced rectal cancer. Lancet.

[12] Heald R., Ryall R. (1986). RECURRENCE AND SURVIVAL AFTER TOTAL MESORECTAL EXCISION FOR RECTAL CANCER. Lancet.

[13] Krook J.E., Moertel C.G., Gunderson L.L., Wieand H.S., Collins R.T., Beart R.W., Kubista T.P., Poon M.A., Meyers W.C., Mailliard J.A. (1991). Effective Surgical Adjuvant Therapy for High-Risk Rectal Carcinoma. N. Engl. J. Med..

[14] Trial S.R.C., Cedermark B., Dahlberg M., Glimelius B., Påhlman L., Rutqvist L.E., Wilking N. (1997). Improved Survival with Preoperative Radiotherapy in Resectable Rectal Cancer. N. Engl. J. Med..

[15] Kapiteijn E., Marijnen C.A., Nagtegaal I.D., Putter H., Steup W.H., Wiggers T., Rutten H.J., Pahlman L., Glimelius B., Van Krieken J.H. (2001). Preoperative Radiotherapy Combined with Total Mesorectal Excision for Resectable Rectal Cancer. N. Engl. J. Med..

[16] Bosset J.-F., Calais G., Mineur L., Maingon P., Stojanovic-Rundic S., Bensadoun R.-J., Bardet E., Beny A., Ollier J.-C., Bolla M. (2014). Fluorouracil-based adjuvant chemotherapy after preoperative chemoradiotherapy in rectal cancer: Long-term results of the EORTC 22921 randomised study. Lancet Oncol..

[17] Sauer R., Becker H., Hohenberger W., Rodel C., Wittekind C., Fietkau R., Martus P., Tschmelitsch J., Hager E., Hess C.F. (2004). Preoperative versus Postoperative Chemoradiotherapy for Rectal Cancer. N. Engl. J. Med..

[18] Sauer R., Liersch T., Merkel S., Fietkau R., Hohenberger W., Hess C., Becker H., Raab H.-R., Villanueva M.-T., Witzigmann H. (2012). Preoperative Versus Postoperative Chemoradiotherapy for Locally Advanced Rectal Cancer: Results of the German CAO/ARO/AIO-94 Randomized Phase III Trial After a Median Follow-Up of 11 Years. J. Clin. Oncol..

[19] Bujko K., Nowacki M.P., Nasierowska-Guttmejer A., Michalski W., Bebenek M., Kryj M. (2006). Long-term results of a randomized trial comparing preoperative short-course radiotherapy with preoperative conventionally fractionated chemoradiation for rectal cancer. Br. J. Surg..

[20] Ciseł B., Pietrzak L., Michalski W., Wyrwicz L., Rutkowski A., Kosakowska E., Cencelewicz A., Spałek M., Polkowski W., Jankiewicz M. (2019). Long-course preoperative chemoradiation versus 5 × 5 Gy and consolidation chemotherapy for clinical T4 and fixed clinical T3 rectal cancer: Long-term results of the randomized Polish II study. Ann. Oncol..

[21] Bregni G., Telli T.A., Camera S., Deleporte A., Moretti L., Bali A., Liberale G., Holbrechts S., Hendlisz A., Sclafani F. (2020). Adjuvant chemotherapy for rectal cancer: Current evidence and recommendations for clinical practice. Cancer Treat. Rev..

[22] Sainato A., Nunzia V.C.L., Valentini V., De Paoli A., Maurizi E.R., Lupattelli M., Aristei C., Vidali C., Conti M., Galardi A. (2014). No benefit of adjuvant Fluorouracil Leucovorin chemotherapy after neoadjuvant chemoradiotherapy in locally advanced cancer of the rectum (LARC): Long term results of a randomized trial (I-CNR-RT). Radiother. Oncol..

[23] Breugom A.J., van Gijn W., Muller E.W., Berglund Å., Broek C.B.M.v.D., Fokstuen T., Gelderblom H., Kapiteijn E., Leer J.W.H., Marijnen C.A.M. (2014). Adjuvant chemotherapy for rectal cancer patients treated with preoperative (chemo)radiotherapy and total mesorectal excision: A Dutch Colorectal Cancer Group (DCCG) randomized phase III trial. Ann. Oncol..

[24] Breugom A.J., Swets M., Bosset J.-F., Collette L., Sainato A., Cionini L., Glynne-Jones R., Counsell N., Bastiaannet E., Broek C.B.M.v.D. (2015). Adjuvant chemotherapy after preoperative (chemo)radiotherapy and surgery for patients with rectal cancer: A systematic review and meta-analysis of individual patient data. Lancet Oncol..

[25] Valk M.J.M., Hilling D.E., Bastiaannet E., Meershoek-Klein Kranenbarg E., Beets G.L., Figueiredo N.L., Habr-Gama A., Perez R.O., Renehan A.G., van de Velde C.J.H. (2018). Long-term outcomes of clinical complete responders after neoadjuvant treatment for rectal cancer. Inter-national Watch & Wait Database (IWWD): An international multicentre registry study. Lancet.

[26] Habr-Gama A., Perez R.O., Nadalin W., Sabbaga J., Ribeiro U., Silva e Sousa A.H., Campos F.G., Kiss D.R., Gama-Rodrigues J. (2004). Operative versus nonoperative treatment for stage 0 distal rectal cancer following chemoradiation therapy: Long-term results. Ann. Surg..

[27] Habr-Gama A., Perez R.O., Proscurshim I., Campos F.G., Nadalin W., Kiss D., Gama-Rodrigues J. (2006). Patterns of Failure and Survival for Nonoperative Treatment of Stage c0 Distal Rectal Cancer Following Neoadjuvant Chemoradiation Therapy. J. Gastrointest. Surg..

[28] Habr-Gama A., Gama-Rodrigues J., Julião G.P.S., Proscurshim I., Sabbagh C., Lynn P.B., Perez R.O. (2014). Local Recurrence After Complete Clinical Response and Watch and Wait in Rectal Cancer After Neoadjuvant Chemoradiation: Impact of Salvage Therapy on Local Disease Control. Int. J. Radiat. Oncol..

[29] Maas M., Beets-Tan R.G., Lambregts D.M., Lammering G., Nelemans P.J., Engelen S.M., van Dam R.M., Jansen R.L., Sosef M., Leijtens J.W. (2011). Wait-and-See Policy for Clinical Complete Responders After Chemoradiation for Rectal Cancer. J. Clin. Oncol..

[30] Renehan A.G., Malcomson L., Emsley R., Gollins S., Maw A., Myint A.S., Rooney P.S., Susnerwala S., Blower A., Saunders M.P. (2016). Watch-and-wait approach versus surgical resection after chemoradiotherapy for patients with rectal cancer (the OnCoRe project): A propensity-score matched cohort analysis. Lancet Oncol..

[31] Sanford N.N., Dee E.C.B., Ahn C., Kazmi S.A., Beg M.S., Folkert M.R., Aguilera T.A., Polanco P.M., Pogacnik J.S., Sher D.J. (2020). Recent Trends and Overall Survival of Young Versus Older Adults with Stage II to III Rectal Cancer Treated With and Without Surgery in the United States, 2010–2015. Am. J. Clin. Oncol..

[32] Smith J.J., Strombom P., Chow O.S., Roxburgh C.S., Lynn P., Eaton A., Widmar M., Ganesh K., Yaeger R., Cercek A. (2019). Assessment of a Watch-and-Wait Strategy for Rectal Cancer in Patients with a Complete Response After Neoadjuvant Therapy. JAMA Oncol..

[33] Khrizman P., Niland J.C., ter Veer A., Milne D., Dunn K.B., Carson W.E., Engstrom P.F., Shibata S., Skibber J.M., Weiser M.R. (2013). Postoperative Adjuvant Chemotherapy Use in Patients with Stage II/III Rectal Cancer Treated with Neoadjuvant Therapy: A National Comprehensive Cancer Network Analysis. J. Clin. Oncol..

[34] Fernández-Martos C., Pericay C., Aparicio J., Salud A., Safont M., Massuti B., Vera R., Escudero P., Maurel J., Marcuello E. (2010). Phase II, randomized study of concomitant chemoradiotherapy followed by surgery and adjuvant capecitabine plus oxaliplatin (CAPOX) compared with induction CAPOX followed by concomitant chemoradiotherapy and surgery in magnetic resonance imaging-defined, locally advanced rectal cancer: Grupo cancer de recto 3 study. J. Clin. Oncol..

[35] Glynne-Jones R., Counsell N., Quirke P., Mortensen N., Maraveyas A., Meadows H.M., Ledermann J., Sebag-Montefiore D. (2014). Chronicle: Results of a randomised phase III trial in locally advanced rectal cancer after neoadjuvant chemoradiation randomising postoperative adjuvant capecitabine plus oxaliplatin (XELOX) versus control. Ann. Oncol..

[36] Chau I., Brown G., Cunningham D., Tait D., Wotherspoon A., Norman A.R., Tebbutt N., Hill M., Ross P.J., Massey A. (2006). Neoadjuvant Capecitabine and Oxaliplatin Followed by Synchronous Chemoradiation and Total Mesorectal Excision in Magnetic Resonance Imaging-Defined Poor-Risk Rectal Cancer. J. Clin. Oncol..

[37] Cercek A., Roxburgh C.S., Strombom P., Smith J.J., Temple L.K., Nash G.M., Guillem J.G., Paty P.B., Yaeger R., Stadler Z.K. (2018). Adoption of Total Neoadjuvant Therapy for Locally Advanced Rectal Cancer. JAMA Oncol..

[38] Van der Valk M.J.M., Marijnen C.A.M., van Etten B., Dijkstra E.A., Hilling D.E., Kranenbarg E.M., Putter H., Roodvoets A.G.H., Bahadoer R.R., Fokstuen T. (2020). Compliance and tolerability of short-course radiotherapy followed by preoperative chemotherapy and surgery for high-risk rectal cancer—Results of the international randomized RAPIDO-trial. Radiother. Oncol..

[39] Bahadoer R.R., Dijkstra E.A., van Etten B., Marijnen C.A.M., Putter H., Kranenbarg E.M.-K., Roodvoets A.G.H., Nagtegaal I.D., Beets-Tan R.G.H., Blomqvist L.K. (2021). Short-course radiotherapy followed by chemotherapy before total mesorectal excision (TME) versus preoperative chemoradiotherapy, TME, and optional adjuvant chemotherapy in locally advanced rectal cancer (RAPIDO): A randomised, open-label, phase 3 trial. Lancet Oncol..

[40] Jin J., Tang Y., Hu C., Jiang L.M., Jiang J., Li N., Liu W.Y., Chen S.L., Li S., Lu N. (2022). Multicenter, Randomized, Phase III Trial of Short-Term Radiotherapy Plus Chemotherapy Versus Long-Term Chemoradiotherapy in Locally Advanced Rectal Cancer (STELLAR). J. Clin. Oncol..

[41] Bujko K., Wyrwicz L., Rutkowski A., Malinowska M., Pietrzak L., Kryński J., Michalski W., Olędzki J., Kuśnierz J., Zając L. (2016). Long-course oxaliplatin-based preoperative chemoradiation versus 5 × 5 Gy and consolidation chemotherapy for cT4 or fixed cT3 rectal cancer: Results of a randomized phase III study. Ann. Oncol..

[42] Chen L.-N., Jiang J., Jiang L.-M., Zhou H.-T., Li N., Lu N.-N., Gao Y.-H., Liu S.-X., Wang W.-L., Wei L.-C. (2024). Post-hoc analysis of clinicopathological factors affecting lateral lymph node metastasis based on STELLAR study for rectal cancer. Radiother. Oncol..

[43] Zhang Y., Tang Y., Ma H., Su H., Xu Z., Gao C., Zhou H., Jin J. (2024). Number of lymph nodes retrieved in patients with locally advanced rectal cancer after total neoadjuvant therapy: Post-hoc analysis from the STELLAR trial. BJS Open.

[44] Jimenez-Fonseca P., Salazar R., Valenti V., Msaouel P., Carmona-Bayonas A. (2022). Is short-course radiotherapy and total neoadjuvant therapy the new standard of care in locally advanced rectal cancer? A sensitivity analysis of the RAPIDO clinical trial. Ann. Oncol..

[45] Dijkstra E.A., Nilsson P.J., Hospers G.A.P., Bahadoer R.R., Meershoek-Klein Kranenbarg E., Roodvoets A.G.H., Putter H., Berglund Å., Cervantes A., Crolla R.M.P.H. (2023). Locoregional Failure During and After Short-course Radiotherapy Followed by Chemotherapy and Surgery Compared with Long-course Chemoradiotherapy and Surgery: A 5-Year Follow-up of the RAPIDO Trial. Ann. Surg..

[46] Conroy T., Bosset J.F., Etienne P.L., Rio E., François É., Mesgouez-Nebout N., Vendrely V., Artignan X., Bouché O., Gargot D. (2021). Neoadjuvant chemotherapy with FOLFIRINOX and preoperative chemoradiotherapy for patients with locally advanced rectal cancer (UNICANCER-PRODIGE 23): A multicentre, randomised, open-label, phase 3 trial. Lancet Oncol..

[47] Conroy T., Castan F., Etienne P.-L., Rio E., Mesgouez-Nebout N., Evesque L., Vendrely V., Artignan X., Bouché O., Gargot D. (2024). Total neoadjuvant therapy with mFOLFIRINOX versus preoperative chemoradiotherapy in patients with locally advanced rectal cancer: Long-term results of the UNICANCER-PRODIGE 23 trial. Ann. Oncol..

[48] Scott A.J., Kennedy E.B., Berlin J., Brown G., Chalabi M., Cho M.T., Cusnir M., Dorth J., George M., Kachnic L.A. (2024). Management of Locally Advanced Rectal Cancer: ASCO Guideline. J. Clin. Oncol..

[49] Wang X., Liu P., Xiao Y., Meng W., Tang Y., Zhou J., Ding P.-R., Ding K.-F., Wang B., Guo Q. (2024). Total neoadjuvant treatment with long-course radiotherapy versus concurrent chemoradiotherapy in local advanced rectal cancer with high risk factors (TNTCRT): A multicenter, randomized, open-label, phase 3 trial. J. Clin. Oncol..

[50] Fokas E., Schlenska-Lange A., Polat B., Klautke G., Grabenbauer G.G., Fietkau R., Kuhnt T., Staib L., Brunner T., Grosu A.L. (2022). Chemoradiotherapy Plus Induction or Consolidation Chemotherapy as Total Neoadjuvant Therapy for Patients with Locally Advanced Rectal Cancer: Long-term Results of the CAO/ARO/AIO-12 Randomized Clinical Trial. JAMA Oncol..

[51] Cerdan-Santacruz C., Julião G.P.S., Vailati B.B., Corbi L., Habr-Gama A., Perez R.O. (2023). Watch and Wait Approach for Rectal Cancer. J. Clin. Med..

[52] Fokas E., Appelt A., Glynne-Jones R., Beets G., Perez R., Garcia-Aguilar J., Rullier E., Smith J.J., Marijnen C., Peters F.P. (2021). International consensus recommendations on key outcome measures for organ preservation after (chemo)radiotherapy in patients with rectal cancer. Nat. Rev. Clin. Oncol..

[53] Garcia-Aguilar J., Patil S., Gollub M.J., Kim J.K., Yuval J.B., Thompson H.M., Verheij F.S., Omer D.M., Lee M., Dunne R.F. (2022). Organ Preservation in Patients with Rectal Adenocarcinoma Treated with Total Neoadjuvant Therapy. J. Clin. Oncol..

[54] Smith J.J., Chow O.S., Gollub M.J., Nash G.M., Temple L.K., Weiser M.R., Guillem J.G., Paty P.B., Avila K., Garcia-Aguilar J. (2015). Organ Preservation in Rectal Adenocarcinoma: A phase II randomized controlled trial evaluating 3-year disease-free survival in patients with locally advanced rectal cancer treated with chemoradiation plus induction or consolida-tion chemotherapy, and total mesorectal excision or nonoperative management. BMC Cancer.

[55] Verheij F.S., Omer D.M., Williams H., Lin S.T., Qin L.-X., Buckley J.T., Thompson H.M., Yuval J.B., Kim J.K., Dunne R.F. (2024). Long-Term Results of Organ Preservation in Patients with Rectal Adenocarcinoma Treated with Total Neoadjuvant Therapy: The Randomized Phase II OPRA Trial. J. Clin. Oncol..

[56] Beets N.R.A.M., Verheij F.S., Williams H., Omer D.M., Lin S.T., Qin L.-X., Beets G.L., Beets-Tan R.G.H., Wei I.H., Widmar M. (2024). Association of Lateral Pelvic Lymph Nodes with Disease Recurrence and Organ Preservation in Patients with Distal Rectal Adenocarcinoma Treated with Total Neoadjuvant Therapy. Ann. Surg..

[57] Thompson H.M., Omer D.M., Lin S., Kim J.K., Yuval J.B., Verheij F.S., Qin L.X., Gollub M.J., Wu A.J., Lee M. (2024). Organ Preservation and Survival by Clinical Response Grade in Patients with Rectal Cancer Treated with Total Neoadjuvant Therapy: A Secondary Analysis of the OPRA Randomized Clinical Trial. JAMA Netw. Open.

[58] Williams H., Fokas E., Diefenhardt M., Lee C., Verheij F., Omer D., Lin S., Dunne R., Marcet J., Cataldo P. (2025). Survival among patients treated with total mesorectal excision or selective watch-and-wait after total neoadjuvant therapy: A pooled analysis of the CAO/ARO/AIO-12 and OPRA randomized phase II trials. Ann. Oncol..

[59] Alvarez J.A., Shi Q., Dasari A., Garcia-Aguilar J., Sanoff H., George T.J., Hong T., Yothers G., Philip P., Nelson G. (2024). Alliance A022104/NRG-GI010: The Janus Rectal Cancer Trial: A randomized phase II/III trial testing the efficacy of triplet versus doublet chemotherapy regarding clinical complete response and disease-free survival in patients with locally advanced rectal cancer. BMC Cancer.

[60] Rullier E., Vendrely V., Asselineau J., Rouanet P., Tuech J.-J., Valverde A., de Chaisemartin C., Rivoire M., Trilling B., Jafari M. (2020). Organ preservation with chemoradiotherapy plus local excision for rectal cancer: 5-year results of the GRECCAR 2 randomised trial. Lancet Gastroenterol. Hepatol..

[61] Bach S.P., STAR-TREC Collaborative (2022). Can we Save the rectum by watchful waiting or TransAnal surgery following (chemo)Radiotherapy versus Total mesorectal excision for early REctal Cancer (STAR-TREC)? Protocol for the international, multicentre, rolling phase II/III partially randomized patient preference trial evaluating long-course concurrent chemoradio-therapy versus short-course radiotherapy organ preservation approaches. Color. Dis..

[62] OncoDaily STAR-TREC Study Shows successful Organ Preservation with Chemoradiotherapy or Short-Course Radiotherapy, Reducing the Need for Surgery in Early-Intermediate Stage Rectal Cancer Patients. https://oncodaily.com/societies/star-trec-estro-2025-press-release.

[63] Jensen L., Poulsen L., Risum S., Nielsen J., Mynster T., Ploeen J., Rahr H., Havelund B., Appelt A., Lindebjerg J. (2020). 400MO Curative chemoradiation for low rectal cancer: Early clinical outcomes from a multicentre phase II trial. Ann. Oncol..

[64] Arp D.T., Appelt A.L., Jensen L.H., Havelund B.M., Nissen H.D., Risumlund S.L., Sjölin M.E.E., Nielsen M.S., Poulsen L.Ø. (2024). Treatment planning for patients with low rectal cancer in a multicenter prospective organ preservation study. Phys. Medica.

[65] Gerard J.-P., Barbet N., Schiappa R., Magné N., Martel I., Mineur L., Deberne M., Zilli T., Dhadda A., Myint A.S. (2023). Neoadjuvant chemoradiotherapy with radiation dose escalation with contact x-ray brachytherapy boost or external beam radiotherapy boost for organ preservation in early cT2–cT3 rectal adenocarcinoma (OPERA): A phase 3, randomised controlled trial. Lancet Gastroenterol. Hepatol..

[66] Benezery K., Montagne L., Evesque L., Schiappa R., Hannoun-Levi J.-M., Francois E., Thamphya B., Gerard J.-P. (2020). Clinical response assessment after contact X-Ray brachytherapy and chemoradiotherapy for organ preservation in rectal cancer T2-T3 M0: The time/dose factor influence. Clin. Transl. Radiat. Oncol..

[67] Wang H., Zhang X., Leng B., Zhu K., Jiang S., Feng R., Dou X., Shi F., Xu L., Yue J. (2024). Efficacy and safety of MR-guided adaptive simultaneous integrated boost radiotherapy to primary lesions and positive lymph nodes in the neoadjuvant treatment of locally advanced rectal cancer: A randomized controlled phase III trial. Radiat. Oncol..

[68] Deng Y., Chi P., Lan P., Wang L., Chen W., Cui L., Chen D., Cao J., Wei H., Peng X. (2019). Neoadjuvant Modified FOLFOX6 With or Without Radiation Versus Fluorouracil Plus Radiation for Locally Advanced Rectal Cancer: Final Results of the Chinese FOWARC Trial. J. Clin. Oncol..

[69] Zhang J., Chi P., Shi L., Cui L., Gao J., Li W., Wei H., Cheng L., Huang Z., Cai G. (2025). Neoadjuvant Modified Infusional Fluorouracil, Leucovorin, and Oxaliplatin With or Without Radiation Versus Fluorouracil Plus Radiation for Locally Advanced Rectal Cancer: Updated Results of the FOWARC Study After a Median Follow-Up of 10 Years. J. Clin. Oncol..

[70] Schrag D., Shi Q., Weiser M.R., Gollub M.J., Saltz L.B., Musher B.L., Goldberg J., Al Baghdadi T., Goodman K.A., McWilliams R.R. (2023). Preoperative Treatment of Locally Advanced Rectal Cancer. N. Engl. J. Med..

[71] Basch E., Dueck A.C., Mitchell S.A., Mamon H., Weiser M., Saltz L., Gollub M., Rogak L., Ginos B., Mazza G.L. (2023). Patient-Reported Outcomes During and After Treatment for Locally Advanced Rectal Cancer in the PROSPECT Trial (Alliance N1048). J. Clin. Oncol..

[72] Mei W.-J., Wang X.-Z., Li Y.-F., Sun Y.-M., Yang C.-K., Lin J.-Z., Wu Z.-G., Zhang R., Wang W., Li Y. (2022). Neoadjuvant Chemotherapy with CAPOX Versus Chemoradiation for Locally Advanced Rectal Cancer with Uninvolved Mesorectal Fascia (CONVERT): Initial Results of a Phase III Trial. Ann. Surg..

[73] Ding P.-R., Wang X.-Z., Li Y.-F., Sun Y.-M., Yang C.-K., Wu Z.-G., Zhang R., Wang W., Zhuang Y.-Z., Lei J. (2023). LBA26 Neoadjuvant chemotherapy with CAPOX versus chemoradiation for locally advanced rectal cancer with uninvolved mesorectal fascia (CONVERT): Final results of a phase III trial. Ann. Oncol..

[74] Ruppert R., Kube R., Strassburg J., Lewin A., Baral J., Maurer C.A., Sauer J., Junginger T., Hermanek P., Merkel S. (2020). Avoidance of Overtreatment of Rectal Cancer by Selective Chemoradiotherapy: Results of the Optimized Surgery and MRI-Based Multimodal Therapy Trial. J. Am. Coll. Surg..

[75] Group M.S. (2006). Diagnostic accuracy of preoperative magnetic resonance imaging in predicting curative resection of rectal cancer: Prospective observational study. Br. Med. J..

[76] Kennedy E.D., Simunovic M., Jhaveri K., Kirsch R., Brierley J., Drolet S., Brown C., Vos P.M., Xiong W., MacLean T. (2019). Safety and Feasibility of Using Magnetic Resonance Imaging Criteria to Identify Patients with “Good Prognosis” Rectal Cancer Eligible for Primary Surgery: The Phase 2 Nonrandomized QuickSilver Clinical Trial. JAMA Oncol..

[77] Li J., Hu Y.-T., Liu C.-C., Wang L.-H., Ju H.-X., Huang X.-F., Chi P., Du J.-L., Wang J.-P., Xiao Y. (2024). Primary Surgery Followed by Selective Chemoradiotherapy Versus Preoperative Chemoradiotherapy Followed by Surgery for Locally Advanced Rectal Cancer: A Randomized Clinical Trial. Int. J. Radiat. Oncol..

[78] He W., Huang J., Liao G., Li W., Shen J., Fu Y., Tang N., He F., Zhao Y., Liu Z. (2025). Short-course radiation(SCRT) followed by 6 cycles of cadonilimab plus mFOLFOX6 as neoadjuvant therapy for patients with locally advanced rectal cancer (LARC): A multicenter, single arm, phase II trial (NeoCaCRT). J. Clin. Oncol..

[79] Shamseddine A., Turfa R., Chehade L., Zeidan Y.H., El Husseini Z., Kreidieh M., Bouferraa Y., Elias C., Kattan J., Khalifeh I. (2025). Short-course radiation followed by mFOLFOX-6 plus avelumab for locally-advanced microsatellite stable rectal adenocarcinoma: The Averectal study. Eur. J. Cancer.

[80] Yang L., Cui X., Wu F., Chi Z., Xiao L., Wang X., Liang Z., Li X., Yu Q., Lin X. (2024). The efficacy and safety of neoadjuvant chemoradiotherapy combined with immunotherapy for locally advanced rectal cancer patients: A systematic review. Front. Immunol..

[81] Thong D.W., Chakraborty P., Rajan R., Theophilus M. (2025). Adjuvant Radiotherapy in Incidental Positive Nodal Disease in Rectal Cancer—A Systemic Review. J. Surg. Oncol..

[82] Polamraju P., Haque W., Verma V., Wiederhold L., Hatch S., Butler E.B., Teh B.S. (2018). Adjuvant Management of Pathologic Node–Positive Disease After Definitive Surgery for Clinical T1-2 N0 Rectal Cancer. Clin. Color. Cancer.

[83] Peng J., Li X., Ding Y., Shi D., Wu H., Cai S. (2013). Is adjuvant radiotherapy warranted in resected pT1-2 node-positive rectal cancer?. Radiat. Oncol..

[84] Akeel N., Lan N., Stocchi L., Costedio M.M., Dietz D.W., Gorgun E., Kalady M.F., Karagkounis G., Kessler H., Remzi F.H. (2017). Clinically Node Negative, Pathologically Node Positive Rectal Cancer Patients Who Did Not Receive Neoad-juvant Therapy. J. Gastrointest. Surg..

[85] Huh J.W., Lim S.W., Kim H.R., Kim Y.J. (2011). Effects of Postoperative Adjuvant Radiotherapy on Recurrence and Survival in Stage III Rectal Cancer. J. Gastrointest. Surg..

[86] Kariv Y., Kariv R., Hammel J.P., Lavery I.C. (2008). Postoperative Radiotherapy for Stage IIIA Rectal Cancer: Is It Justified?. Dis. Colon Rectum.

[87] Komori K., Kimura K., Kinoshita T., Sano T., Ito S., Abe T., Senda Y., Misawa K., Ito Y., Uemura N. (2014). Complications Associated with Postoperative Adjuvant Radiation Therapy for Advanced Rectal Cancer. Int. Surg..

[88] Pilar A., Gupta M., Laskar S.G., Laskar S. (2017). Intraoperative radiotherapy: Review of techniques and results. ecancermedicalscience.

[89] Haddock M.G. (2017). Intraoperative radiation therapy for colon and rectal cancers: A clinical review. Radiat. Oncol..

[90] Amarnath S.R. (2023). The Role of Intraoperative Radiotherapy Treatment of Locally Advanced Rectal Cancer. Clin. Colon Rectal Surg..

[91] Liu B., Ge L., Wang J., Chen Y.-Q., Ma S.-X., Ma P.-L., Zhang Y.-Q., Yang K.-H., Cai H. (2020). Efficacy and safety of intraoperative radiotherapy in rectal cancer: A systematic review and meta-analysis. World J. Gastrointest. Oncol..

[92] Tom M.C., Joshi N., Vicini F., Chang A.J., Hong T.S., Showalter T.N., Chao S.T., Wolden S., Wu A.J., Martin D. (2019). The American Brachytherapy Society consensus statement on intraoperative radiation therapy. Brachytherapy.

[93] Calvo F.A., Sole C.V., Rutten H.J., Poortmans P., Asencio J.M., Serrano J., Aristu J., Roeder F., Dries W.J. (2020). ESTRO/ACROP IORT recommendations for intraoperative radiation therapy in primary locally advanced rectal cancer. Clin. Transl. Radiat. Oncol..

